# CD169 Expressing Macrophage, a Key Subset in Mesenteric Lymph Nodes Promotes Mucosal Inflammation in Dextran Sulfate Sodium-Induced Colitis

**DOI:** 10.3389/fimmu.2017.00669

**Published:** 2017-06-26

**Authors:** Qiuting Li, Dan Wang, Shengyu Hao, Xiaolei Han, Yuan Xia, Xiangzhi Li, Yaoxing Chen, Masato Tanaka, Chun-Hong Qiu

**Affiliations:** ^1^Department of Cell Biology, Shandong University School of Medicine, Jinan, Shandong, China; ^2^Laboratory of Veterinary Anatomy, College of Animal Medicine and Agricultural University, Beijing, China; ^3^Laboratory of Immune Regulation, School of Life Science, Tokyo University of Pharmacy and Life Sciences, Hachioji, Tokyo, Japan

**Keywords:** mesenteric lymph nodes, CD169^+^ macrophages, dextran sulfate sodium, colitis, cytokines

## Abstract

Inflammatory bowel disease (IBD) including Crohn’s disease (CD) and ulcerative colitis is a relapsing-remitting illness. Patients with long-standing extensive colitis are easy to develop colorectal cancer (CRC). The increasing incidence of IBD and a substantial increase in the risk of CRC make the necessity to pay more attention on the regulation of inflammation especially by specific macrophages subset. The present study reported that a key subset of sinus macrophage expressing CD169 in mesenteric lymph nodes (mLNs) played an essential role in promoting mucosal inflammation. The results revealed that the subset expressing CD169 in mLNs increased significantly during the dextran sulfate sodium (DSS)-induced colitis. The colitic symptoms were alleviated in CD169-diphtheria toxin receptor (DTR) mice at least partially due to the deletion of CD169^+^ macrophages in mLNs. In addition, the levels of inflammatory cytokines as well as the percentage of Th17 cells in mLNs from CD169-DTR mice were much lower than those from WT mice with DSS-induced colitis. Further experiment *in vitro* demonstrated that the supernatant from whole cells of mLNs or colon tissues could promote the production of inflammatory factors by mLN cells or colon tissues from CD169-DTR mice. These results could be explained by the cell sorting result that CD11b^+^CD169^+^ macrophages expressed higher level of inflammatory factors directly. All these data indicated that CD169^+^ sinus macrophage in mLNs played an essential role on regulating mucosal inflammation.

## Introduction

Inflammatory bowel disease (IBD), which includes ulcerative colitis (UC) or Crohn’s disease (CD), has been paid more attention due to their increasing incidence and a substantial increase in the risk of colorectal cancer (CRC). IBD is called a disease of developed countries as its association with industrialization of nations, and its high incidence rates and prevalence in North America and Europe (1). However, the incidence of IBD is increasing with time in different regions around the world, such as East Asia and North Africa, indicating its emergence as a worldwide disease (2, 3). IBD is a relapsing-remitting illness, patients with long-standing extensive colitis develop colorectal cancer (CRC) easily (4, 5). Although the disease pathogenesis is not fully understood, some various components including environmental risk factors, gut microbiota, genetic susceptibility, and components of the immune response, such as the innate and adaptive immune system, are implicated in the pathogenesis of IBD (6, 7). Activation of T cells and monocytes/macrophages is regarded as a crucial factor in the pathogenesis of IBD (8–11).

Macrophages are an important group involved in innate immunity, due to existence in most of the tissues around the body (12). At sites of infection, macrophages encounter and engulf invading microbes. The various anatomical localization of macrophage is mirrored by their substantial phenotypic diversity and plasticity (13). Over the past few years, there has been a great deal of interests in deciphering the essential roles of macrophages for local homeostasis, a balance between inflammation and protective immunity.

CD169, also known as sialoadhesin or sialic acid binding immunoglobulin-like lectin (Siglec) 1 (14), is strongly expressed by specific macrophage subsets in secondary lymphoid organs, such as subcapsular sinus, medullary macrophages in LN, and marginal metallophilic macrophages in spleen (15). In both secondary lymphoid organs, CD169^+^ macrophages are located at the entry sites of lymph or blood, present as gatekeepers at these sites where antigens enter and perform an activation of T and B cells and antipathogen immune response (16). In the colon, CD169^+^ macrophages are mainly located in the lamina propria, and its number increased significantly during colonic inflammation when mice were supplied with dextran sulfate sodium (DSS) in drinking water for 7 days (16, 17). This macrophage population produced CCL8 in response to epithelial injury, thus initiating mucosal inflammation by recruiting inflammatory monocytes (17). In lymph node, due to their strategic position, CD169^+^ macrophages can serve as cellular “flypaper,” filter the pathogens when tissue-derived lymph pass through one or more draining LNs, preventing systemic pathogen dissemination (18–20). Some reports have showed that mesenteric lymph nodes (mLNs) are important inductive sites and critical regulators in mucosal inflammation (21, 22); however, the mechanism of sinus macrophages in mLNs that regulate the mucosal inflammation remains elusive.

The gut-associated lymphoid tissues can be divided into effector sites, and organized inductive sites. Inductive sites, such as Peyer’s patches (PPs), mLNs, and isolated lymphoid follicles, are responsible for the induction phase of the mucosal immune responses (21). It has been reported that intestinal dendritic cells (DCs) mature and transport from the lamina propria and PPs to the draining mLNs after antigen uptake, where they encounter and prime naïve T cells into specific help (Th) cells, such as Th1, Th2, or the recently identified Th17 cells (22). These colitogenic effector cells then exit the mLNs via the efferent lymphatic vessels, enter into the systemic circulation, and migrate from them LNs to the gut, where the effector cells initiate intestinal inflammation (23). Emerging evidence suggests that IL-17 plays a crucial role in colon inflammation and carcinogenesis (24), the gut inflammation occurring in IBD patients is also characterized by the production of cytokines made by Th17 cells, a distinct lineage of Th cells (25). It would be essential to understand the mechanism of mucosal inflammation by revealing the roles of CD169 expressing cells on Th17 differentiation in mLNs.

It is necessary to elucidate the critical mechanism of IBD in order to prevent further inflammation progression. The present study aims to reveal the role of mLN CD169^+^ macrophages in mucosal inflammation regulation by using CD169-diphtheria toxin receptor (DTR) mice. It is indicated that sinus macrophage subset expressing CD169 in mLNs could serve as a key subset, promoting the progress of mucosal inflammation in DSS-induced colitis, probably by secreting higher levels of inflammatory cytokines. This report will provide novel clues for clinical medicines.

## Materials and Methods

### Mice

C57BL/6 mice were purchased from Vital River Laboratories, Beijing, and were bred in our specific pathogen-free animal facility. CD169-DTR mice were provided by the RIKEN BioResource Center (RBRC No. 04395) with the approval of depositors (26, 27). These mice were housed at Shandong University Medical School Animal Care Facility. All mice used in this research were matched for age and sex. All research protocols were approved by the Shandong University of the Animal Care and Utilization Committee and approved by the local government authorities.

### DSS-Induced Colitis Model

Female mice received one cycle (3 or 7 days) of 3.5% DSS (MW 5000, Wako, Japan) treatment in the drinking water and their body weight was monitored daily for 7 days from the administration of DSS. Recipient mice used for experiments were between the age of 8 and 12 weeks. For some experiments of macrophage depletion, mice were intraperitoneally injected with 10 µg/kg body weight of diphtheria toxin (DT) (Sigma, MO) 1 day before and 3 days after the administration of DSS.

### Flow Cytometry

Mesenteric lymph node cells were incubated with Fc blocker (clone 93; Biolegend, CA, USA) at ice, then cell-surface staining was performed using the following antibodies: FITC anti-mouse CD11b (M1/70); PE anti-mouse CD169 (3D6.112); and PE anti-mouse CD4(GK1.5). Intracellular staining was performed using the APC anti-mouse IL-17A (TC11-18H10.1). All antibodies were purchased from Biolegend biosciences. For intracellular staining, the above T lymphocytes or polarized naive CD4^+^ cells were first re-stimulated with PMA (100 ng/ml) (Sigma) and ionomycin (750 ng/ml) (Sigma, A23187, USA) in the presence of Golgiplug (BD) for 5 h. Fixation Buffer (Biolegend, 420801) and Intracellular Staining Permeabilization Wash Buffer (Biolegend, 421002) were used for cell fixation and permeabilization.

### Histopathology and Immunohistochemistry

For hematoxylin and eosin (H&E), mLNs and distal colons were routinely fixed in 10% neutral buffered formalin, then embedded in paraffin and stained with hematoxylin and eosin. For immunohistochemistry, mLNs were snap-frozen in Tissue Tek O.C.T. Compound (Sakura, Japan). The 4–5 µm sectioned mLNs were fixed in 4% paraformaldehyde, 10% normal goat serums was used to block non-specific bonding sites for 45 min. Slides then were incubated with FITC anti-mouse CD11b (M1/70); PE anti-mouse CD169 (3D6.112) overnight at 4°C and washed with phosphate buffer (PBS). All the stained slides were observed by fluorescence microscopy (Nikon, Japan) and analyzed using NIS-Elements BR 3.2.

### Cytokine Measurements

Total RNA was extracted from mLNs and distal colons using Trizol Reagent (Invitrogen, CA, USA). Template cDNA was synthesized using the ReverTra Ace qPCR RT kit (Toyobo, Japan). Real-time quantitative PCR was carried out using SYBR green PCR master mix (Toyobo, Japan), β-actin was used as the internal control. The reaction was performed in a CFX96 Real-Time PCR Detection System (Bio-Rad, CA, USA). The sequences of the primers used are the following: IL-17-R: CTCCAGAAGGCCCTCAGACTAC, IL-17-F: AGCTTTCCCTCCGCATTGACACAG, IL-21-R: TGTTTCTTTCCTCCCCTCCT, IL-21-F: ATGCAGCTTTTGCCTGTTTT, IL-23-R: CAGGGAACAAGATGCTGGAT, IL-23-F: GGCTAGCATGCAGAGATTCC, IL-6-R: CTGGAGTACCATAGCTACC, IL-6-F: TGTTAGGAGAGCATTGGA, IL-1β-R: GGATGAGGACATGAGCACCT, IL-1β-F: AGCTCATATGGGTCCGACA, TNFα-R: ACCCTCACACTCAGATCATC, TNFα-F: GAGTAGACAAGGTACAACCC, IL-12-R: AGCAGTAGCAGTTCCCCTGA, IL-12-F: AGTCCCTTTGGTCCAGTGTG, IL-18-R: GGGTTCACTGGCACTTTGAT, IL-18-F: ACAACTTTGGCCGACTTCAC, CCL3-R: GGCATTCAGTTCCAGGTCAG, CCL3-F: TCCCAGCCAGGTGTCATTT, CCL8-R: GCTGTGGTTTTCCAGACCAA, CCL8-F: GAAGGTTCAAGGCTGCAGAA, CCL22-R: CAGGCAGGTCTGGGTGAA, CCL22-F: TAAAGGTGGCGTCGTTGG, CD169-R: CAATTTCCGGTGCTTACGGTG, CD169-F: CATAGTCTAGGCTTCTGTGC, β-actin-R: TGCGTGACATCAAAGAGAAG, β-actin-F: TCCATACCCAAGAAGGAAGG.

### Culture of mLN Cells and Colon Tissues

Mesenteric lymph node cells obtained from WT colitis and CD169-DTR colitis mice were cultured in 6-well plate (Santa Cruz) (1 × 10^7^ cells/well) with 3 mL RPMI 1640 medium containing 10% fetal bovine serum (Gibico). Freshly obtained distal colons were flushed five times with PBS to remove feces. Three-mm long colon tissues (4 biopsy samples/well) were cultured in a 24-well plate (Santa Cruz) with 1 mL RPMI 1640 medium, at 37°C in 5% CO_2_ humidified air for 12 h. Then, the supernatant of the mLNs cells and colons from WT colitis was transferred to mLNs cells or colons from CD169-DTR mice. mLNs cells and colons were cultured for 12 h further, total RNA from the cultured cells and colon tissues were extracted, and levels of IL-1β, IL-21, IL-23, CCL22, CCL3, and CCL8 mRNAs were then analyzed by quantitative real-time PCR.

### Statistical Analysis

Statistical analysis was performed using GraphPad Prism 5.0 (GraphPad Software, San Diego, CA, USA). Data were presented as the mean ± SD and were analyzed by the unpaired *t*-test. Values of *P* < 0.05 were considered significantly.

## Results

### Higher Expression of Cytokines and Chemokines in mLNs during the Colitis Course

**Figure 1:**
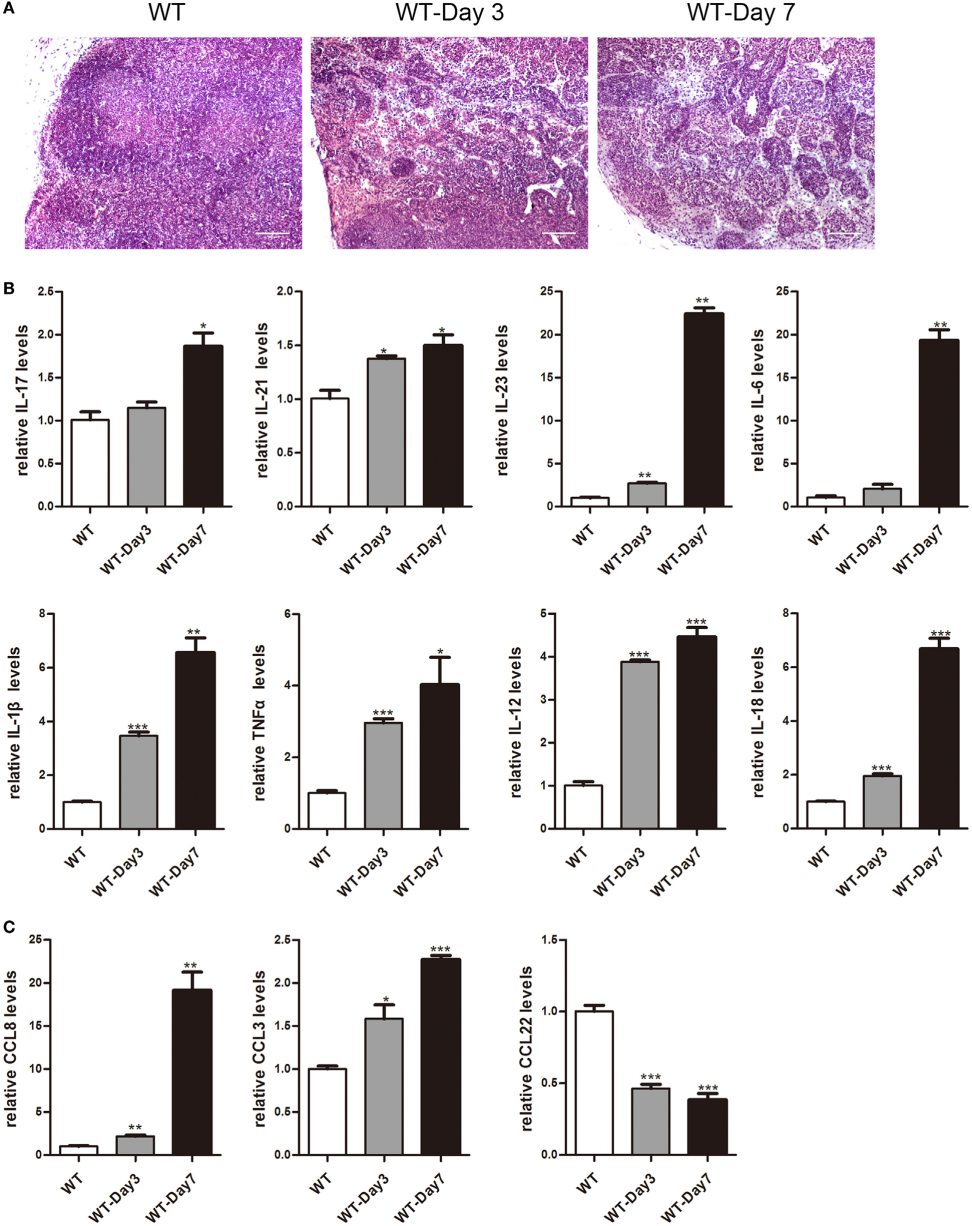
Histopathological changes and cytokines, chemokines expression of mesenteric lymph nodes (mLNs) during the colitis course. C57BL/6 WT mice were treated with 3.5% dextran sulfate sodium (DSS), and mice were harvested after 3 or 7 days. **(A)** Histological analysis of mLN sections obtained from WT control mice and DSS-treated mice (WT-Day3 and WT-Day7). **(B)** Real-time PCR analysis of pro-inflammatory cytokines in WT mice and DSS-treated mice (WT-Day3 and WT-Day7). **(C)** Chemokines expression in mLNs obtained from representative mice from indicated groups. Statistical analysis was determined by Student’s *t*-test, *P < 0.05, **P < 0.01, ***P < 0.001 compared to control. Data are representative of two independent experiments.

It has been reported that mLNs are important inductive sites and critical regulators of epithelial injury-induced mucosal inflammation (21, 23). This study aimed to discover whether mice mLNs were pathogenically relevant for acute inflammation in the gut. 3.5% DSS-treated mice showed a significant decrease in body weight, obvious rectal bleeding, colon shortening, and increase of infiltrated inflammatory cells in the colon (Figure S1 in Supplementary Material). Histological examination revealed that the percentage of infiltrated inflammatory cells significantly increased and the mLNs were characterized by follicular hyperplasia and sinus reaction (Figure 1A). The expression levels of cytokines and chemokines in mLNs from colitic mice were analyzed by real-time PCR (Figures 1B,C). mRNA expression levels of IL-17, IL-21, IL-23, IL-6, IL-1β, TNFα, IL-12, IL-18, CCL8, and CCL3 were much higher in mLN tissues from DSS-treated mice than those from non-treated mice. However, the expression of CCL22, an anti-inflammatory factor (28), was decreased significantly (Figure 1C). These findings suggested that mLNs play an essential role in the pathogenesis of UC.

### CD169^+^ Macrophages Increased in mLNs of Colitis Mice

**Figure 2:**
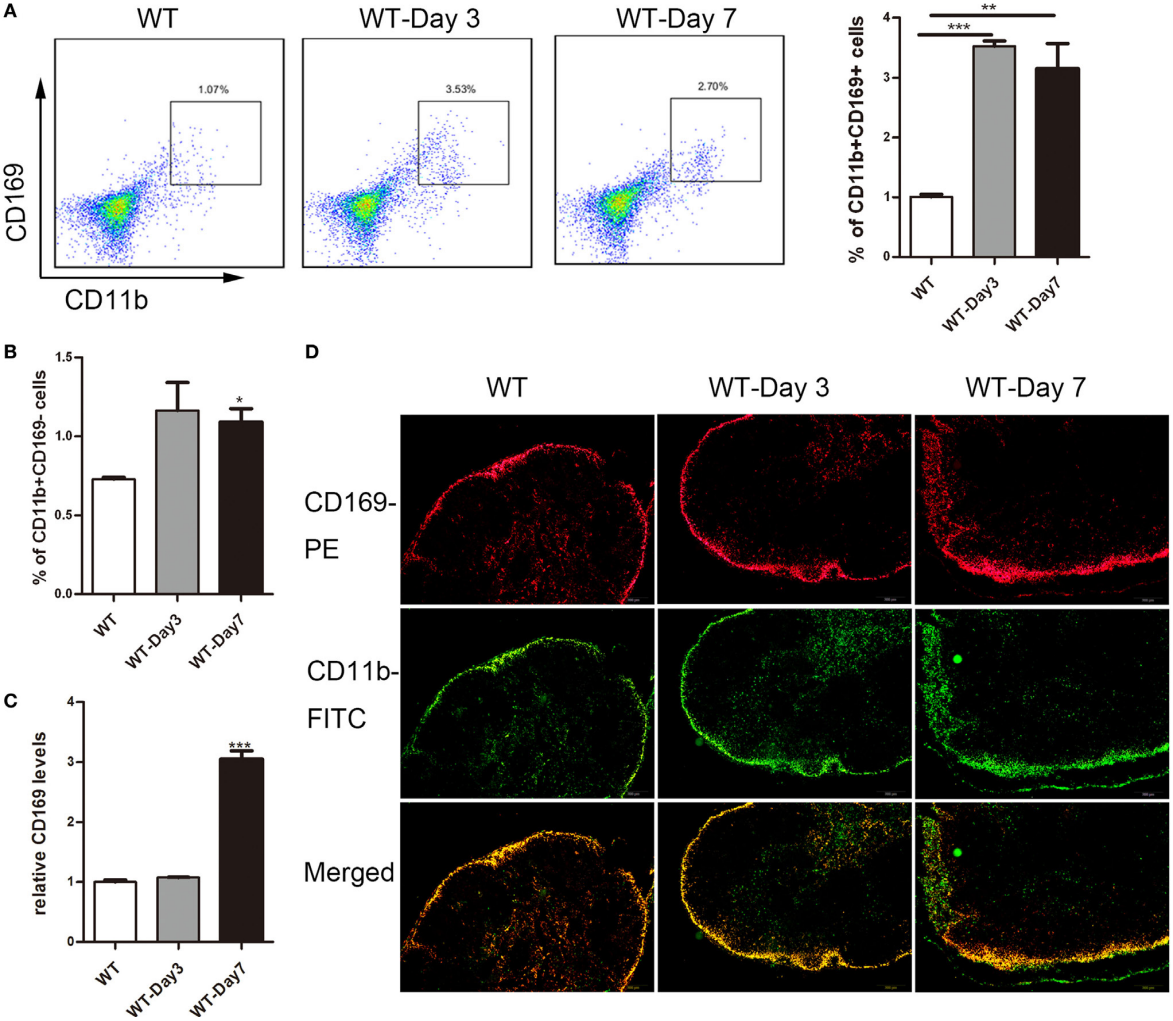
CD169^+^ macrophages are increased in mesenteric lymph nodes (mLNs) of colitis mice. C57BL/6 WT mice were treated with 3.5% dextran sulfate sodium (DSS), and mice were harvested after 3 or 7 days. **(A)** The percentage of CD11b^+^CD169^+^ macrophages in mLNs of WT control mice and DSS-treated mice were measured by FACS, and statistical analysis was shown, *P < 0.05, ***P < 0.001 compared to control. **(B)** The percentage of CD11b^+^CD169^−^ macrophages in mLNs of WT control mice and DSS-treated mice, *P < 0.05 compared to control. **(C)** CD169 mRNA expression in mLNs obtained from WT control mice and DSS-treated mice, **P < 0.01 compared to control. **(D)** Immunohistochemistry analysis of mLN sections obtained from representative mice of indicated groups.

Emerging evidence suggested a crucial role of lamina propria CD169^+^ macrophage in colon inflammation and carcinogenesis (17), which prompted us to explore whether subscapular sinus macrophage expressing CD169 subset in mLNs play its role in IBD further. First, flow cytometry analysis was performed to examine the change of CD169^+^ macrophages in mLNs from DSS-treated mice. The result revealed that about three times of the percentage of CD11b^+^CD169^+^ macrophages significantly increased in mice with daily administration of DSS for 3 and 7 days compared to no treated mice, which was not shown in CD11b^+^CD169^−^ subset (Figures 2A,B). Second, the results of real-time PCR revealed that the expression level of CD169 was much higher in the mLNs of colitic mice (Figure 2C). Finally, the result of immunofluorescence histochemistry showed that CD11b^+^CD169^+^ macrophages subset was mainly localized in the sinus of the mLNs and the number increased from colitic mice compared to control mice (Figure 2D). These data raised the possibility that sinus CD169^+^ macrophage in mLNs would be an important subset to regulate the mucosal inflammation in the pathogenesis of colitis.

### Typical Colitis by DSS Treatment in WT Mice Were Not Observed in CD169-DTR Mice

**Figure 3:**
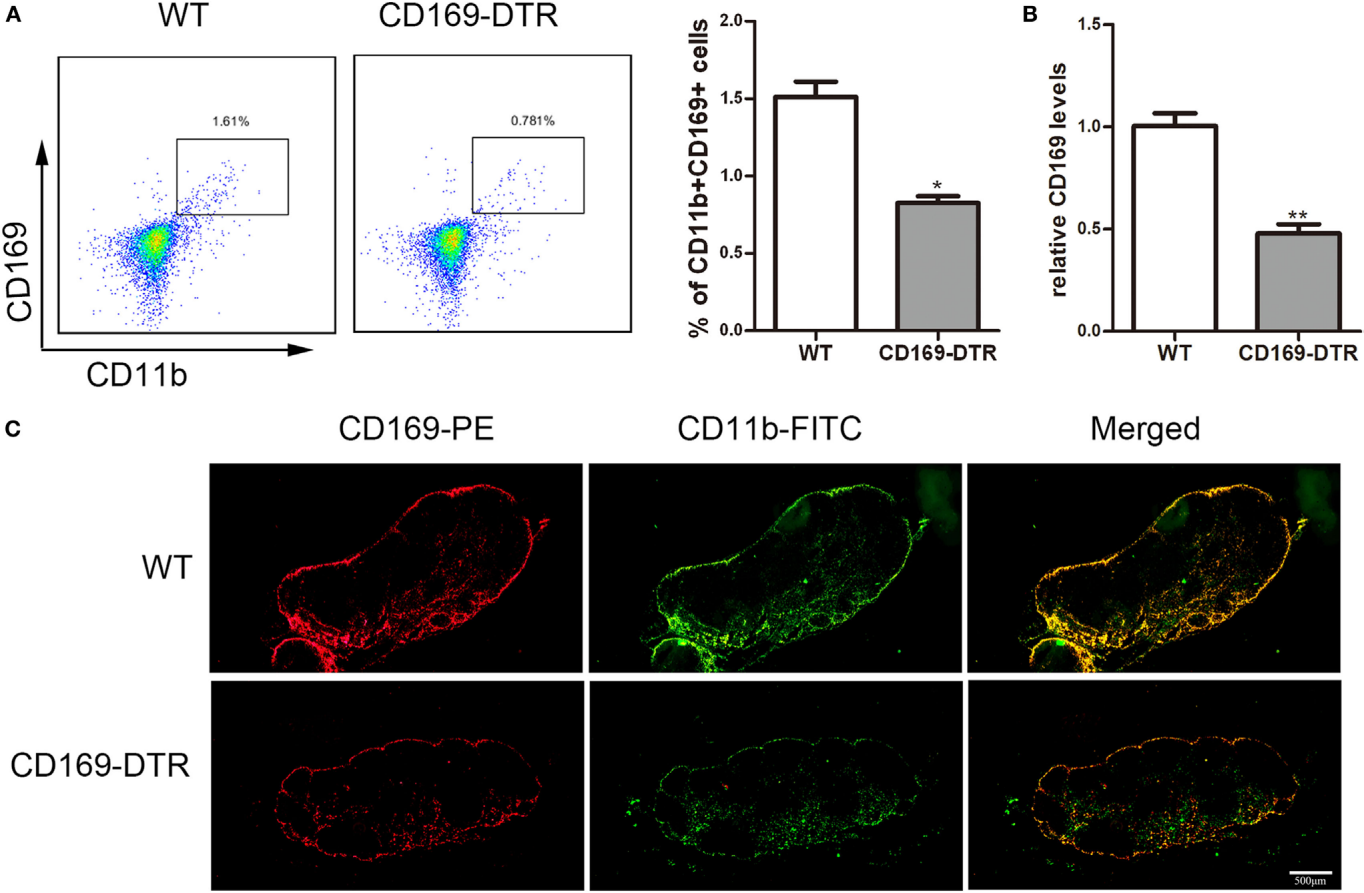
CD11b^+^CD169^+^ macrophages in mesenteric lymph nodes (mLNs) from CD169-diphtheria toxin receptor (DTR) mice was deleted by diphtheria toxin (DT) administration. CD169-DTR mice were intraperitoneally injected with DT (day 0), Mice were harvested on day 3. **(A)** Flow cytometry analysis of CD11b^+^CD169^+^ macrophage in mLNs obtained from WT control mice and CD169-DTR mice with DT treatment, and statistical analysis of the result of FACS, *P < 0.05 compared to control. **(B)** CD169 mRNA expression in mLNs obtained from WT control mice and CD169-DTR mice with DT treatment, **P < 0.01 compared to control. **(C)** mLN sections obtained from representative mice from indicated groups were detected by Immunohistochemistry. Data are representative of two independent experiments.

**Figure 4:**
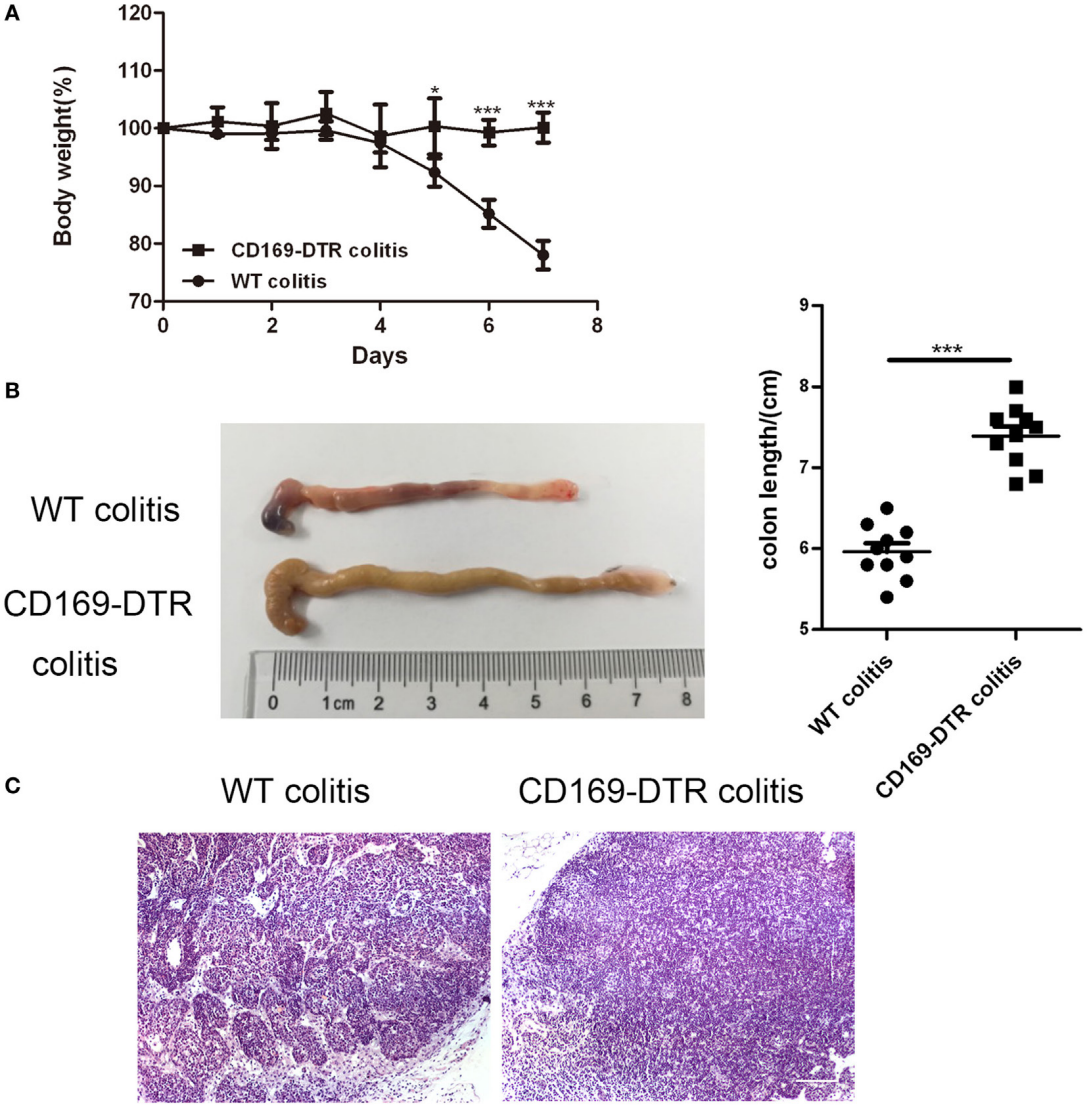
Typical colitis in WT mice caused by dextran sulfate sodium (DSS) treatment were not observed in CD169-diphtheria toxin receptor (DTR) mice. WT and CD169-DTR mice with diphtheria toxin treatment were both orally treated with 3.5% DSS; 7 days later, mice were harvested. **(A)** Weight loss of WT mice (*n* = 5) and CD169-DTR mice (*n* = 5) treated with the indicated time points with DSS, *P < 0.05, ***P < 0.001compared to control. **(B)** Macroscopic observation of colons on day 7, such as the length of colons and bloody stools (left), and the statistical analysis of the length of WT colitis and CD169-DTR colitis mice colons (right) (*n* = 10), ***P < 0.001 compared to control. **(C)** Immunohistochemistry analysis of mesenteric lymph node sections obtained from WT colitis mice and CD169-DTR colitis mice. Data are representative of two independent experiments.

To determine the role of CD169^+^ macrophages in mLNs in the pathogenesis of colitis, CD169-DTR transgenic mice were used during the following study. DT (10 µg/kg) was intraperitoneally administrated into the CD169-DTR mice. Three days later, the change of CD11b^+^CD169^+^ macrophages in mLNs was detected by flow cytometry. The percentage of CD11b^+^CD169^+^ macrophages significantly decreased in CD169-DTR mice compared to WT control mice (Figure 3A). Real-time PCR analysis of mRNA confirmed the lower expression of CD169 in CD169-DTR mice than that in WT mice (Figure 3B). Moreover, immunohistological analysis also showed the decrease in percentage of CD11b^+^CD169^+^ macrophages in the mLNs from CD169-DTR mice compared with that in age-matched wild-type mice (Figure 3C). These data demonstrated that CD169^+^ macrophages in mLNs could be effectively deleted by DT administration in CD169-DTR mice. Next, WT and CD169-DTR mice were both orally treated with 3.5% DSS, and the pathological change of colitis between WT and CD169-DTR mice was compared. Compared with severe colitis in WT mice, DSS-treated CD169-DTR mice showed moderate symptoms, such as loss of body weight, shortening of colon length, and tissue injury (Figures 4A,B). The results clearly showed that typical colitis in WT mice were not observed in CD169-DTR mice. Furthermore, histological examination revealed much less infiltration of inflammatory cells in mLNs from DSS-treated CD169-DTR mice, and relatively little follicular hyperplasia (Figure 4C). Therefore, these results clearly revealed that the severity of DSS-induced colitis could be improved in CD169-DTR mice, at least due to the depletion of CD169^+^ macrophages in mLNs.

### Th17-Related Cytokines and the Percentage of Th17 Cells Could Be Affected by the Presence or Not of CD169^+^ Subset in mLNs

**Figure 5:**
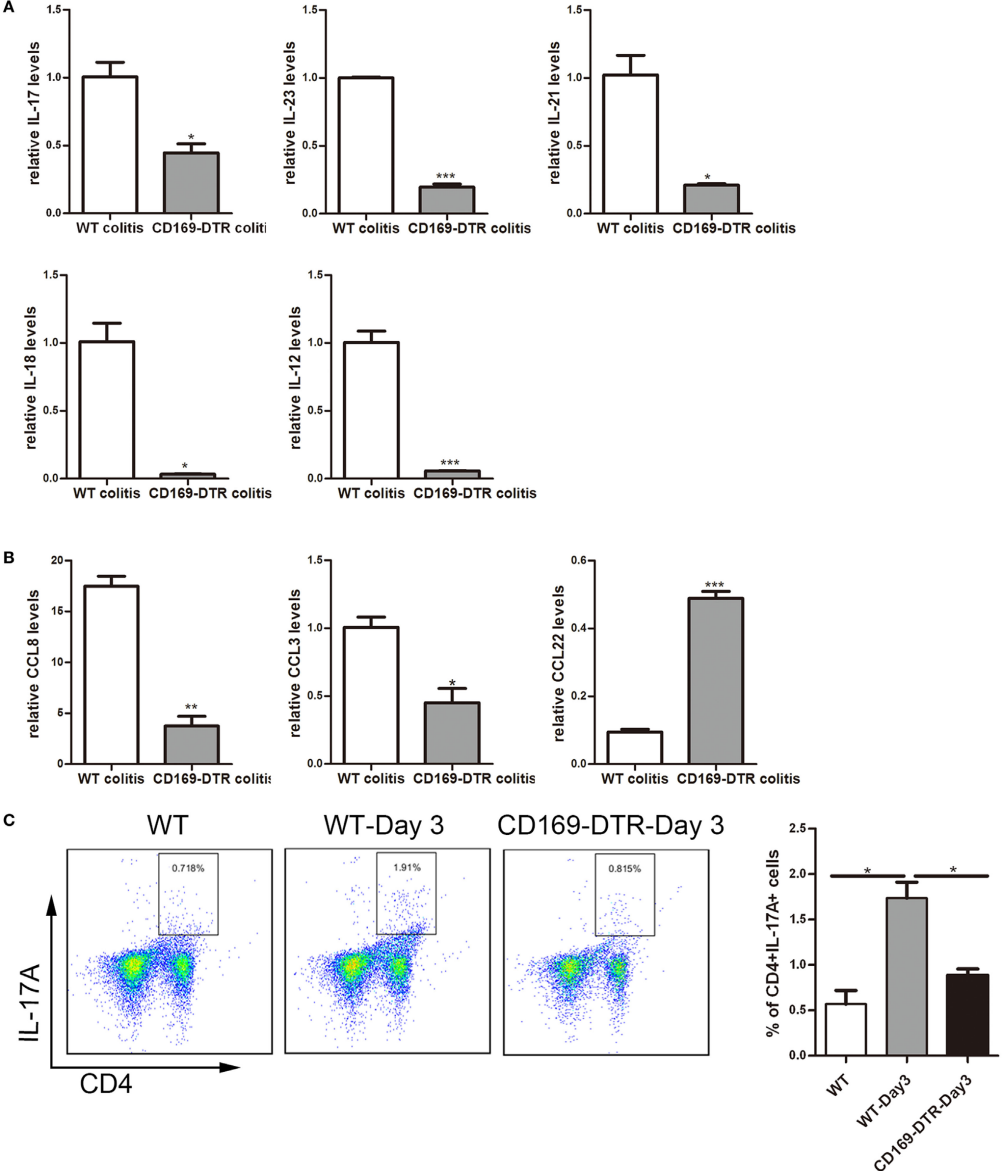
Th17-related cytokines and the percentage of Th17 cells could be affected by the presence or not of CD169^+^ subset in mesenteric lymph nodes (mLNs). WT and CD169-diphtheria toxin receptor (DTR) mice with diphtheria toxin treatment were both orally treated with 3.5% dextran sulfate sodium (DSS) for 7 days. **(A)** Th17-related cytokines expression levels in mLNs. **(B)** Real-time PCR analysis of chemokines in mLNs of DSS-treated WT mice and CD169-DTR mice. **(C)** Flow cytometry analysis of Th17 cells in mLNs obtained from WT control mice, WT colitis mice and CD169-DTR colitis mice and statistical analysis of the result of FACS. *P < 0.05, **P < 0.01, ***P < 0.001 compared to control. Data are representative of two independent experiments.

To obtain cellular and molecular mechanism of the sinus CD169^+^ macrophage-mediated regulation of colonic inflammation, the expression levels of inflammatory cytokines, such as IL-17, IL-23, IL-21, IL-12, and IL-18, as well as chemokines, such as CCL8, CCL3, and CCL22 in mLNs from DSS-treated WT and CD169-DTR mice were examined. mRNA levels of pro-inflammatory cytokines and chemokines, including IL-17, IL-23, IL-21, IL-12, and IL-18, and CCL8 and CCL3 were significantly decreased in CD169-DTR mice. In contrast, the production of CCL22 was significantly increased (Figures 5A,B).

The declined expression of Th17 cell-related cytokines in DT-treated CD169-DTR mice prompted us to investigate whether Th17 cells participating in CD169^+^ macrophage regulating inflammation. Th17 cells from mLNs were analyzed by flow cytometry. The percentage of Th17 cells in DSS-treated CD169-DTR mice was lower than that in WT colitis mice (Figure 5C). These data revealed Th17 cell percentage and Th17-related cytokine levels in CD169-DTR mice could be affected by CD169^+^ subset in mLNs.

### Supernatant of mLNs and Colons from Colitic Mice Could Induce Inflammatory Cytokine Production by mLN Cells and Colons *In Vitro*

**Figure 6:**
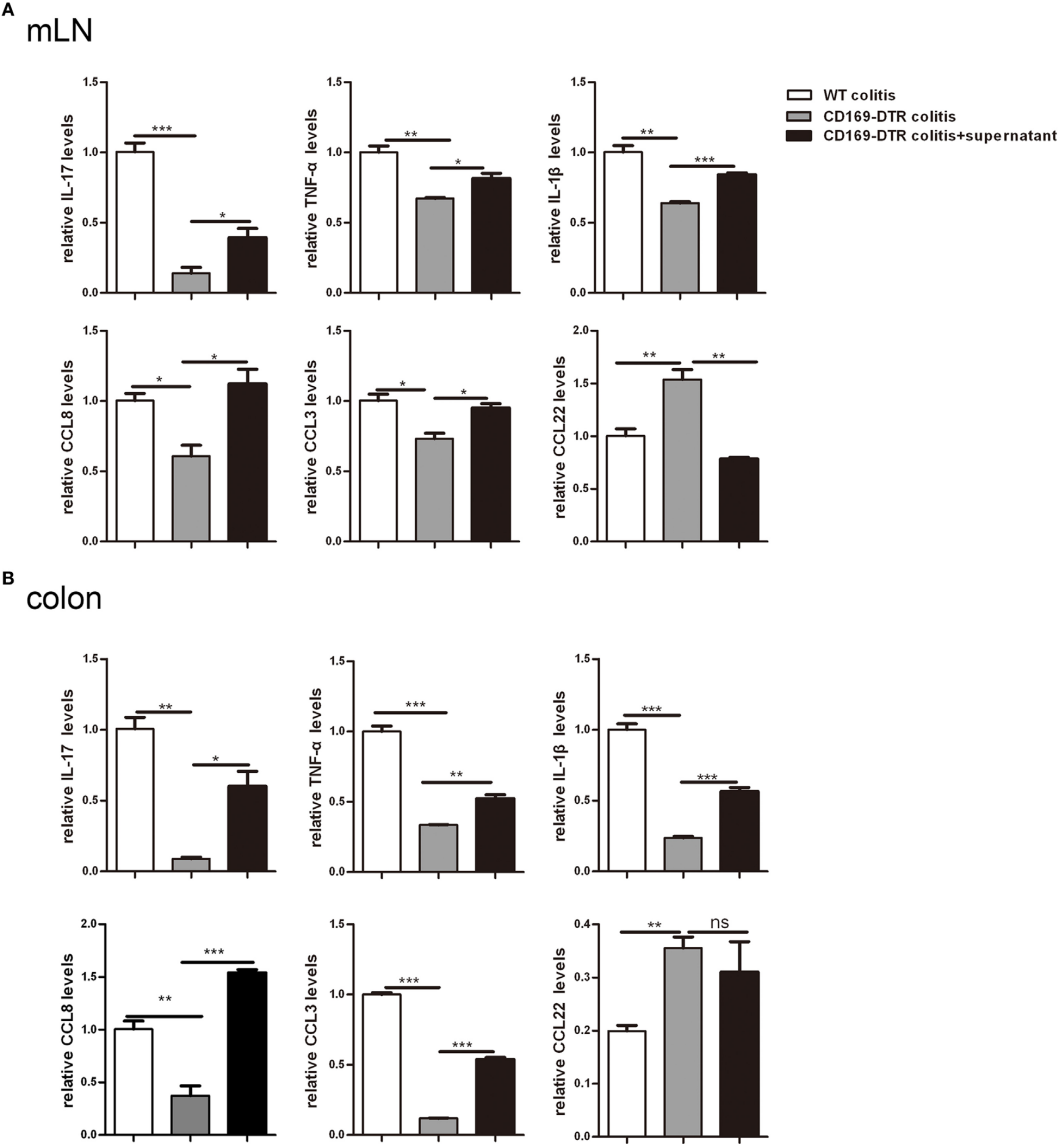
Supernatant of mesenteric lymph nodes (mLNs) and colons from colitic mice could induce inflammatory cytokines production by mLN cells and colons *in vitro*. The supernatant of mLN cells and colons of WT mice with dextran sulfate sodium (DSS) treatment was transferred to the mLN cells and colons of CD169-diphtheria toxin receptor (DTR) mice with DSS treatment, then the cultured cells and colon tissues were harvested. **(A)** Cytokines and chemokines mRNA expression levels of cultured mLNs. **(B)** Cytokines and chemokines mRNA expression levels of cultured colons. *P < 0.05, **P < 0.01, ***P < 0.001 compared to control. Data are representative of two independent experiments.

To further determine the roles of mLNs sinus CD169^+^ macrophages in colonic inflammation, an *in vitro* culture system was developed. mLN cells from WT mice and CD169-DTR mice with DSS treatment were cultured separately *in vitro* for 12 h first, then the supernatant of mLN cells from the WT colitic mice was transferred to cultured cells beforehand from CD169-DTR mice. And all cells were cultured 12 h further. mRNA expression levels of these three kinds of cultured cells were analyzed by real-time PCR. The *in vitro* expression levels of inflammatory cytokines including IL-17, TNFα, and IL-1β and chemokines including CCL8 and CCL3 were lower in mLNs from 169-DTR mice than those from WT mice, which were similar to *in vivo*. However, the expression levels of these factors were enhanced in mLN cells cultured with transferred supernatant of WT colitic mice. However, CCL22 level in mLNs from 169-DTR mice was higher than that from WT mice, and the expression level was inhibited in mLN cells when cultured with supernatant from WT mice (Figure 6A). The same experiment was also performed with cultured colon tissues. The expression levels of all detected factors (Figure 6B) were reversed in colon tissues from CD169-DTR mice cultured with transferred supernatant of WT colitic mice. The data demonstrated that the presence of CD169^+^ macrophages in mLNs and colons could directly regulate inflammatory factors production by mLNs and colons from CD169-DTR mice with CD169^+^ macrophages deletion *in vitro*.

### CD169^+^ Macrophages Secreted Higher Pro-inflammatory Factors Dominantly

**Figure 7:**
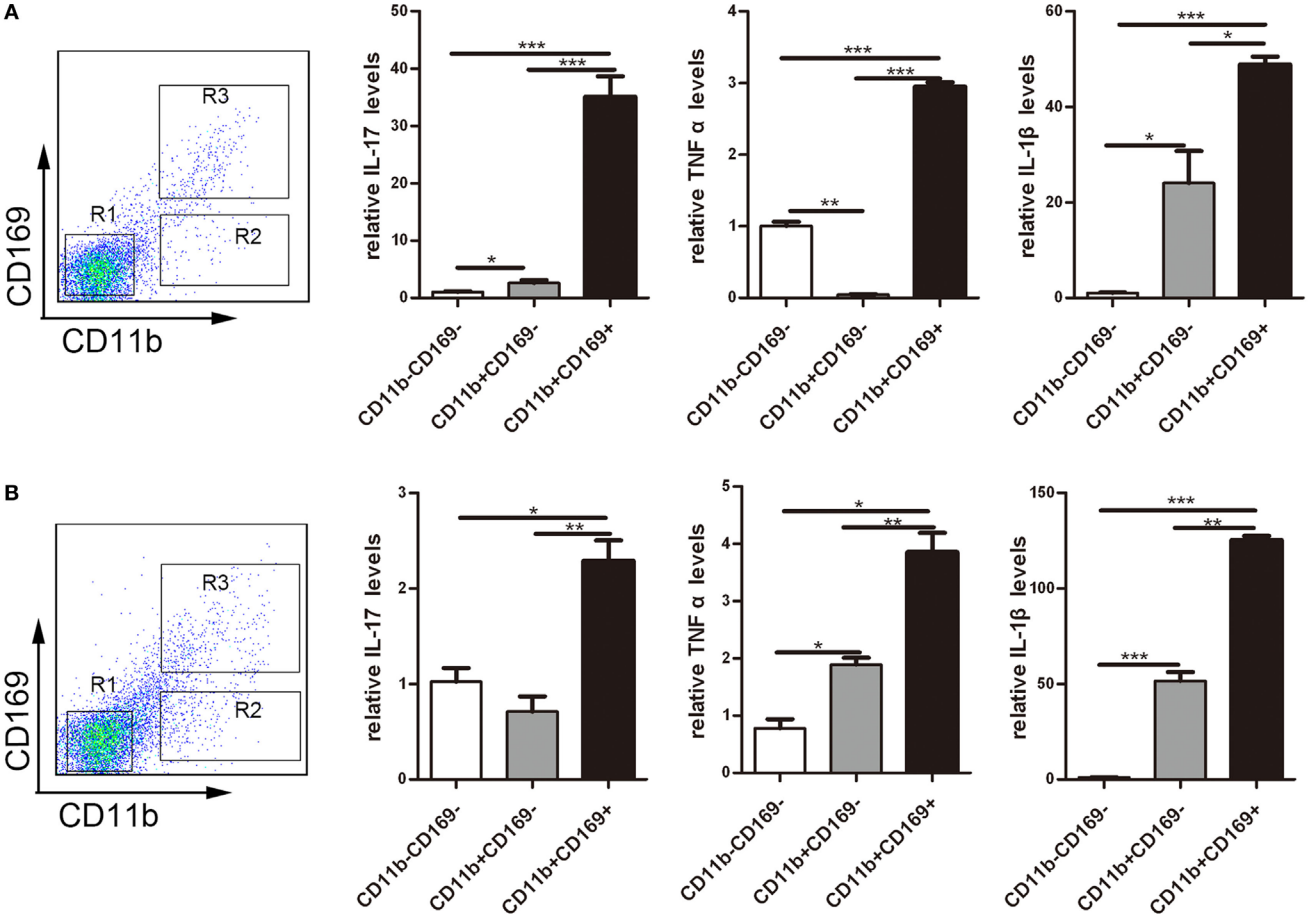
CD169^+^ macrophages can secret pro-inflammatory cytokines and chemokines directly. Mice were orally treated with 3.5% dextran sulfate sodium (DSS) for 7 days, then mesenteric lymph node cells from WT control mice and DSS-treated WT mice were sorted into three subpopulations by flow cytometry. **(A)** Pro-inflammatory factors’ mRNA expression levels of three subpopulations sorted from WT mice. **(B)** Pro-inflammatory factors’ mRNA expression levels of three subpopulations sorted from WT colitis mice, *P < 0.05, **P < 0.01, ***P < 0.001 compared to control. Data are representative of two independent experiments.

To understand the role of CD169^+^ cells promoting mucosal inflammation clearly, the expression profiles of inflammatory factors were compared with the three sorted subpopulations: R1: CD11b^−^CD169^−^ macrophages, R2: CD11b^−^CD169^+^ macrophages, and R3: CD11b^+^CD169^+^ macrophages from mLNs of WT mice. Real-time PCR analysis of these cells revealed that CD11b^+^CD169^+^ macrophages expressed much higher pro-inflammatory factors, whereas the other two subpopulations showed intermediate or lower expression level (Figure 7A). These data suggested that the CD11b^+^CD169^+^ macrophages could secrete higher level of IL-17, TNFα, and IL-1β than the other two subsets in mLNs. Expression patterns of three subpopulations from mLNs of WT colitis mice were also compared, the mRNA expression levels of IL-17, TNFα, and IL-1β were also much higher in CD11b^+^CD169^+^ macrophages than those in the other two subpopulations (Figure 7B). It is suggested that CD11b^+^CD169^+^ macrophage in mLNs had the capacity to secrete pro-inflammatory cytokines directly.

## Discussion

Inflammatory bowel disease including UC and CD is an idiopathic disease caused by a deregulated immune response to host intestinal microflora (29). Macrophages play an essential role for intestinal homeostasis and the pathology of IBD; recent studies have demonstrated that distinct macrophages populations of intestinal are responsible for their functional plasticity under different conditions (30). Studies about how intestinal macrophages play a significant role; however, distinct roles in healthy and inflamed intestine should to be continued and CD169^+^ macrophages have been the focus of research in recent years (17, 31).

LNs are secondary lymphoid organs thst are crucial for the initiation of adaptive immune responses, and mLNs are an important site of T-cell activation for the colon (32). The results of this paper collectively demonstrated a very important function of mLNs CD169^+^ macrophages in DSS-induced colitis. CD11b^+^CD169^+^ macrophages were significantly increased in mLNs of WT mice after daily administration with DSS compared to non-treated mice, and typical colitic symptoms were not observed in CD169-DTR mice with deletion of CD169^+^ cells by DT injection. Th17-related cytokines and the percentage of Th17 cells were significantly decreased in CD169-DTR mice compared to those in WT colitis mice. CD11b^+^CD169^+^ macrophages could secret pro-inflammatory factors directly, which may promote the mucosal inflammation *in vitro*. These results demonstrated that CD169^+^ macrophages in mLNs were an important subset regulating mucosal inflammation.

Many researchers have been paying close attention to studies on CD169^+^ macrophages for many years, because of their unique distribution, redistribution upon immune activation, and their contribution to antigen handling (33). In spleen, the deletion of CD169^+^ macrophages enhanced lymphocyte responsiveness to apoptotic cell antigens and accelerated disease progression in an animal model of systemic lupus erythematous (34). The selective CCL22 expression by CD169^+^ macrophage located in splenic marginal zone is sufficient to drive induction of long-term suppression to apoptotic cell antigens by rapid recruitment of Tregs (35). In colon, CD169^+^ macrophage mainly located in laminar propria could produce CCL8 in response to epithelial injury to initiate colitis by recruiting inflammatory monocytes (17). Many studies have revealed that mLNs can efficiently accommodate naive lymphocytes to encounter antigens, control the development, migration, and functional differentiation of immune cells of the adaptive immune system (36–38), innate cells including macrophages and DCs play important roles during these immune processes by bringing antigens from intestinal tissues to mLNs. Due to its strategic position, CD169^+^ macrophage in mLNs can rapidly encounter pathogens, antigens, and exosomes reaching the LNs through afferent lymph.

This study was performed based on the hypothesis that CD169^+^ macrophage in mLNs would be an important subset in mucosal inflammation. The study showed that the number of CD169^+^ macrophage in mLNs of WT mice increased in DSS-induced colitis, and CD11b^+^CD169^+^ subset produced higher expression levels of pro-inflammatory factors than the other two subsets in mLNs. It has been demonstrated that these cells have an important role in cell–cell communication (39, 40). All these reports strongly support the hypothesis that CD169 expressing cells in mLNs were important for mucosal inflammation. However, the precise mechanism by which CD169^+^ macrophages promote the development of colitis remains unclear.

Among multiple mechanisms involved in the pathogenesis of colitis, innate and adaptive immunity both play crucial roles. Th17 cells have been implicated as important mediators of inflammation in IBD (9, 41). The combination of IL-6 plus transforming growth factor-β are sufficient to induce differentiation of Th17 cells from naive T cells and IL-23 can trigger the proliferation of Th17 cells from activated memory T cells. It also has been shown that IL-6 can drive the expression of IL-21 in Th17 cells and IL-21 in turn can serve as an autocrine cytokine that is sufficient and necessary for Th17 differentiation (42–44). IL-17 and IL-17F as well as IL-6, GM-CSF, and TNF can be produced by Th17 cells; some reports demonstrated that IL-12 and IL-10 family member can also be secreted by Th17 cells (42, 45, 46). The data in this paper revealed that the expression levels of IL-17, IL-21, IL-23, IL-6, IL-1β, TNFα, and IL-12 in mLN tissues significantly decreased in CD169-DTR colitis mice compared to those in WT colitis mice. Many studies demonstrated that Th17 cells were involved in the pathogenesis of IBD and experimental colitis, and mLNs can play a crucial role in initiating the intestinal inflammation as well as dictate quantitative and qualitative features of the response (45, 47). The results of this study showed that the percentage of Th17cells decreased in mLNs from CD169-DTR mice compared to that in WT mice treated with DSS. This revealed that CD169^+^ macrophages in mLNs were essential for Th17 cell-mediated colitis probably through regulating the production of Th17-related cytokines. Among those detected cytokines, IL-17 is mainly produced by Th17 cells, Th17 cells also can secrete other cytokines, such as IL-21, TNFα, and IL-1β, moreover, IL-6 and IL-23, but little IL-12, which are the most efficient inducers of Th17 cells differentiation (22, 25). The concrete mechanism for these novel observations need to be explored further.

This study successfully demonstrated that the production of inflammatory factors by mLNs cells or colon tissues from CD169-DTR mice with deletion of CD169 cells could be reversed by supernatant from whole cells of mLNs or colons. The present results suggested that the production of inflammatory factors promoting mucosal inflammation in WT mice is related to the presence of CD169 cells or not. It is highly necessary to do an effort to identify the key factor produced by these cells on regulating the mucosal inflammation.

In summary, our data reported new findings in the pathogenesis of colitis: first, the number of CD11b^+^CD169^+^ macrophages in mLNs significantly increased in the DSS-induced colitic mice. Second, the typical colitis symptom was not induced in CD169-DTR mice treated with DSS, and the levels of inflammatory cytokines as well as the percentage of Th17 cells in mLNs from CD169-DTR mice were much lower than those from WT mice with DSS-induced colitis, which at least partially due to the deletion of CD169^+^ macrophages. Third, further experiment *in vitro* demonstrated that the supernatant from whole cells of mLNs or colon tissues could promote the production of inflammatory factors by mLN cells or colon tissues from CD169-DTR mice. These could be explained by the cell sorting result that CD11b^+^CD169^+^ macrophages expressed higher level of inflammatory factors directly. All these data strongly revealed the essential roles of CD169 expressing subset in mLNs on regulating mucosal inflammation.

## Ethics Statement

This study was carried out in accordance with the recommendations of Utilization Committee of Shandong University. All research protocols were approved by the Shandong University of the Animal Care and Utilization Committee and approved by the local government authorities.

## Author Contributions

C-HQ and QL designed experiments and contributed to the writing of the manuscript; QL DW, SH, XH, and YX did experiments; QL, XL, and YC analyzed data; MT provided the CD169-DTR mice and suggestion for the article.

## Conflict of Interest Statement

The authors declare that the research was conducted in the absence of any commercial or financial relationships that could be construed as a potential conflict of interest.

## References

[1] Legaki E Gazouli M. Influence of environmental factors in the development of inflammatory bowel diseases. World J Gastrointest Pharmacol Ther (2016) 7(1):112–25. 10.4292/wjgpt.v7.i1.112 26855817 PMC4734944

[2] Turid H Nielsen KR Munkholm P Burisch J Lynge E. The Faroese IBD study: incidence of inflammatory bowel diseases across 54 years of population-based data. J Crohns Colitis (2016) 10(8):934–42. 10.1093/ecco-jcc/jjw050 26933031 PMC4962362

[3] Molodecky NA Soon IS Rabi DM Ghali WA Ferris M Chernoff G Increasing incidence and prevalence of the inflammatory bowel diseases with time, based on systematic review. Gastroenterology (2012) 142(1):46–54. 10.1053/j.gastro.2011.10.001 22001864

[4] De Arcangelis A Hamade H Alpy F Normand S Bruyere E Lefebvre O Hemidesmosome integrity protects the colon against colitis and colorectal cancer. Gut (2016):1–13. 10.1136/gutjnl-2015-310847 27371534 PMC5595104

[5] Feagins LA Souza RF Spechler SJ. Carcinogenesis in IBD: potential targets for the prevention of colorectal cancer. Nat Rev Gastroenterol Hepatol (2009) 6(5):297–305. 10.1038/nrgastro.2009.44 19404270

[6] Kinugasa T Akagi Y. Status of colitis-associated cancer in ulcerative colitis. World J Gastrointest Oncol (2016) 8(4):351–7. 10.4251/wjgo.v8.i4.351 27096030 PMC4824713

[7] Ray K Steatohepatitis: PARP inhibition protective against alcoholic steatohepatitis and NASH. Nat Rev Gastroenterol Hepatol (2017) 14(1):3 10.1038/nrgastro.2016.186 27848963

[8] Vucelic B. Inflammatory bowel diseases: controversies in the use of diagnostic procedures. Dig Dis (2009) 27(3):269–77. 10.1159/000228560 19786751

[9] Liu Z Cao AT Cong Y. Microbiota regulation of inflammatory bowel disease and colorectal cancer. Semin Cancer Biol (2013) 23(6):543–52. 10.1016/j.semcancer.2013.09.002 24071482 PMC3836974

[10] Medina-Contreras O Geem D Laur O Williams IR Lira SA Nusrat A CX3CR1 regulates intestinal macrophage homeostasis, bacterial translocation, and colitogenic Th17 responses in mice. J Clin Invest (2011) 121(12):4787–95. 10.1172/JCI59150 22045567 PMC3226003

[11] Fujino S Andoh A Bamba S Ogawa A Hata K Araki Y Increased expression of interleukin 17 in inflammatory bowel disease. Gut (2003) 52:65–70. 10.1136/gut.52.1.65 12477762 PMC1773503

[12] Hume DA The mononuclear phagocyte system. Curr Opin Immunol (2006) 18(1):49–53. 10.1016/j.coi.2005.11.008 16338128

[13] Price JV Vance RE The macrophage paradox. Immunity (2014) 41(5):685–93. 10.1016/j.immuni.2014.10.015 25517611

[14] Taylor PR Martinez-Pomares L Stacey M Lin HH Brown GD Gordon S. Macrophage receptors and immune recognition. Annu Rev Immunol (2005) 23:901–44. 10.1146/annurev.immunol.23.021704.115816 15771589

[15] Saunderson SC Dunn AC Crocker PR McLellan AD CD169 mediates the capture of exosomes in spleen and lymph node. Blood (2014) 123:208–16. 10.1182/blood-201303-489732 24255917 PMC3888287

[16] Hiemstra IH Beijer MR Veninga H Vrijland K Borg EG Olivier BJ The identification and developmental requirements of colonic CD169 (^+^) macrophages. Immunology (2014) 142(2):269–78. 10.1111/imm.12251 24883436 PMC4008234

[17] Asano K Takahashi N Ushiki M Monya M Aihara F Kuboki E Intestinal CD169(^+^) macrophages initiate mucosal inflammation by secreting CCL8 that recruits inflammatory monocytes. Nat Commun (2015) 6:7802. 10.1038/ncomms8802 26193821 PMC4518321

[18] Kuka M Iannacone M The role of lymph node sinus macrophages in host defense. Ann N Y Acad Sci (2014) 1319(1):38–46. 10.1111/nyas.12387 24592818

[19] Junt T Moseman EA Iannacone M Massberg S Lang PA Boes M Subcapsular sinus macrophages in lymph nodes clear lymph-borne viruses and present them to antiviral B cells. Nature (2007) 450(7166):110–4. 10.1038/nature06287 17934446

[20] Asano K Nabeyama A Miyake Y Qiu CH Kurita A Tomura M CD169-positive macrophages dominate antitumor immunity by crosspresenting dead cell-associated antigens. Immunity (2011) 34(1):85–95. 10.1016/j.immuni.2010.12.011 21194983

[21] Makita S Kanai T Nemoto Y Totsuka T Okamoto R Tsuchiya K Intestinal lamina propria retaining CD4^+^CD25^+^ regulatory T cells is a suppressive site of intestinal inflammation. J Immunol (2007) 178(8):4937–46. 10.4049/jimmunol.178.8.4937 17404275

[22] Sakuraba A Sato T Kamada N Kitazume M Sugita A Hibi T. Th1/Th17 immune response is induced by mesenteric lymph node dendritic cells in Crohn’s disease. Gastroenterology (2009) 137(5):1736–45. 10.1053/j.gastro.2009.07.09 19632232

[23] Takebayashi K Koboziev I Ostanin DV Gray L Karlsson F Robinson-Jackson SA Role of the gut-associated and secondary lymphoid tissue in the induction of chronic colitis. Inflamm Bowel Dis (2011) 17(1):268–78. 10.1002/ibd.21447 20812332 PMC3072787

[24] Kathania M Khare P Zeng M Cantarel B Zhang H Ueno H Itch inhibits IL-17-mediated colon inflammation and tumorigenesis by ROR-γt ubiquitination. Nat Immunol (2016) 17(8):997–1004. 10.1038/ni.3488 27322655

[25] Galvez J. Role of Th17 cells in the pathogenesis of human IBD. ISRN Inflamm (2014) 2014:928461. 10.1155/2014/928461 25101191 PMC4005031

[26] Saito M Iwawaki T Taya C Yonekawa H Noda M Inui Y Diphtheria toxin receptor-mediated conditional and targeted cell ablation in transgenic mice. Nat Biotechnol (2001) 19(8):746–50. 10.1038/90795 11479567

[27] Miyake Y Asano K Kaise H Uemura M Nakayama M Tanaka M. Critical role of macrophages in the marginal zone in the suppression of immune responses to apoptotic cell-associated antigens. J Clin Invest (2007) 117(8):2268–78. 10.1172/JCI31990 17657313 PMC1924497

[28] Hao S Han X Wang D Yang Y Li Q Li X Critical role of CCL22/CCR4 axis in the maintenance of immune homeostasis during apoptotic cell clearance by splenic CD8alpha(^+^) CD103(^+^) dendritic cells. Immunology (2016) 148(2):174–86. 10.1111/imm.12596 26868141 PMC4863574

[29] Kaistha A Levine J. Inflammatory bowel disease: the classic gastrointestinal autoimmune disease. Curr Probl Pediatr Adolesc Health Care (2014) 44(11):328–34. 10.1016/j.cppeds.2014.10.003 25499459

[30] Bain CC Scott CL Uronen-Hansson H Gudjonsson S Jansson O Grip O Resident and pro-inflammatory macrophages in the colon represent alternative context-dependent fates of the same Ly6Chi monocyte precursors. Mucosal Immunol (2012) 6(3):498–510. 10.1038/mi.2012.89 22990622 PMC3629381

[31] Ohnishi K Komohara Y Saito Y Miyamoto Y Watanabe M Baba H CD169-positive macrophages in regional lymph nodes are associated with a favorable prognosis in patients with colorectal carcinoma. Cancer Sci (2013) 104(9):1237–44. 10.1111/cas.12212 23734742 PMC7657174

[32] Koboziev I Karlsson F Grisham MB. Gut-associated lymphoid tissue, T cell trafficking, and chronic intestinal inflammation. Ann N Y Acad Sci (2010) 1207(Suppl 1):E86–93. 10.1111/j.1749-6632.2010.05711 20961311 PMC3075575

[33] Martinez-Pomares L Gordon S. CD169^+^ macrophages at the crossroads of antigen presentation. Trends Immunol (2012) 33(2):66–70. 10.1016/j.it.2011.11.001 22192781

[34] McGaha TL Chen Y Ravishankar B van Rooijen N Karlsson MC. Marginal zone macrophages suppress innate and adaptive immunity to apoptotic cells in the spleen. Blood (2011) 117(20):5403–12. 10.1182/blood-2010-11-320028 21444914

[35] Ravishankar B Shinde R Liu H Chaudhary K Bradley J Lemos HP Marginal zone CD169^+^ macrophages coordinate apoptotic cell-driven cellular recruitment and tolerance. Proc Natl Acad Sci U S A (2014) 111(11):4215–20. 10.1073/pnas.1320924111 24591636 PMC3964059

[36] Phan TG Green JA Gray EE Xu Y Cyster JG. Immune complex relay by subcapsular sinus macrophages and noncognate B cells drives antibody affinity maturation. Nat Immunol (2009) 10(7):786–93. 10.1038/ni.1745 19503106 PMC2776777

[37] Zhang J Xu J Zhang RX Zhang Y Ou QJ Li JQ CD169 identifies an activated CD8(^+^) T cell subset in regional lymph nodes that predicts favorable prognosis in colorectal cancer patients. Oncoimmunology (2016) 5(7):e1177690. 10.1080/2162402X.2016.1177690 27622027 PMC5006907

[38] Houston SA Cerovic V Thomson C Brewer J Mowat AM Milling S. The lymph nodes draining the small intestine and colon are anatomically separate and immunologically distinct. Mucosal Immunol (2016) 9(2):468–78. 10.1038/mi.2015.77 26329428

[39] Klaas M Crocker PR. Sialoadhesin in recognition of self and non-self. Semin Immunopathol (2012) 34(3):353–64. 10.1007/s00281-012-0310-3 22450957

[40] O’Neill AS van den Berg TK Mullen GE Sialoadhesin – a macrophage-restricted marker of immunoregulation and inflammation. Immunology (2013) 138(3):198–207. 10.1111/imm.12042 23181380 PMC3573273

[41] Wu W He C Liu C Cao AT Xue X Evans-Marin HL miR-10a inhibits dendritic cell activation and Th1/Th17 cell immune responses in IBD. Gut (2015) 64(11):1755–64. 10.1136/gutjnl-2014-307980 25281418

[42] Bettelli E Oukka M Kuchroo VK. T(H)-17 cells in the circle of immunity and autoimmunity. Nat Immunol (2007) 8:345–50. 10.1038/ni0407-345 17375096

[43] Fina D Sarra M Fantini MC Rizzo A Caruso R Caprioli F Regulation of gut inflammation and Th17 cell response by interleukin-21. Gastroenterology (2008) 134(4):1038–48. 10.1053/j.gastro.2008.01.041 18395085

[44] Nurieva R Yang XO Martinez G Zhang Y Panopoulos AD Ma L Essential autocrine regulation by IL-21 in the generation of inflammatory T cells. Nature (2007) 448(7152):480–3. 10.1038/nature05969 17581589

[45] Feng T Qin H Wang L Benveniste EN Elson CO Cong Y. Th17 cells induce colitis and promote Th1 cell responses through IL-17 induction of innate IL-12 and IL-23 production. J Immunol (2011) 186(11):6313–8. 10.4049/jimmunol.1001454 21531892 PMC3249225

[46] Liang SC Tan XY Luxenberg DP Karim R Dunussi-Joannopoulos K Collins M Interleukin (IL)-22 and IL-17 are coexpressed by Th17 cells and cooperatively enhance expression of antimicrobial peptides. J Exp Med (2006) 203(10):2271–9. 10.1084/jem.20061308 16982811 PMC2118116

[47] Shen W Durum SK Synergy of IL-23 and Th17 cytokines: new light on inflammatory bowel disease. Neurochem Res (2010) 35(6):940–6. 10.1007/s11064-009-0091-9 19915978 PMC7241863

