# Supplementary material for: CD169 Expressing Macrophage, a Key Subset in Mesenteric Lymph Nodes Promotes Mucosal Inflammation in Dextran Sulfate Sodium-Induced Colitis
Source: Front Immunol. 2017 Jun 26;8:669. doi: 10.3389/fimmu.2017.00669 (PMC5483437; doi:10.3389/fimmu.2017.00669)
Supplement: Supplementary file 1 [file presentation_1.pdf]

Supplementary Material

CD169 expressing macrophage, a key subset in mLNs promotes mucosal inflammation in DSS-induced colitis

Qiuting Li[1], Dan Wang[1], Shengyu Hao[1], Xiaolei Han[1] Yuan Xia[1], Xiangzhi Li[1],

Yaoxing Chen[2], Masato Tanaka[3], Chun-Hong Qiu†

*** Correspondence:** Chun-Hong Qiu, E-mail: qiuchun@sdu.edu.cn;

1 Supplementary Figures

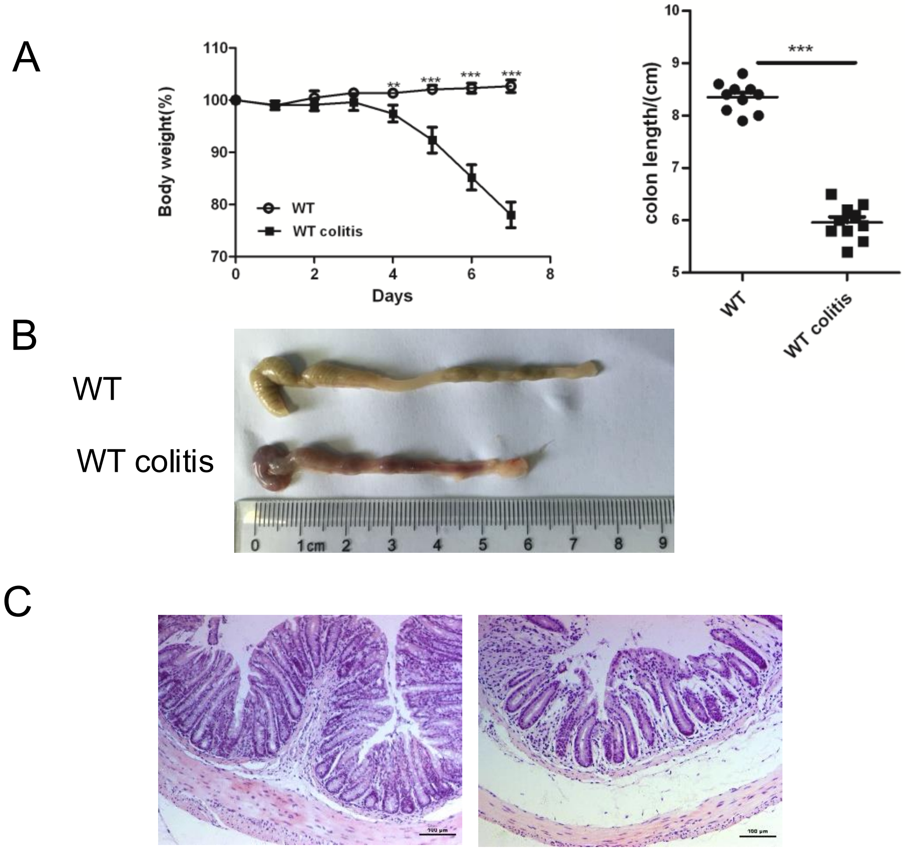

Supplementary Figure 1. WT mice were both orally treated with 3.5%DSS for 7 days. (A)Weight loss of WT mice (n = 5) and WT colitis mice (n=5), **P<0.001.***P<0.001, compared to control. (B) Macroscopic observation of WT control and WT colitis colons and the statistical analysis of the

length of WT control and WT colitis mice colons. (n=10).Statistical analysis was determined by Student's t test,***P<0.001. (C)Immunohistochemistry analysis of colons sections obtained from WT control mice and WT colitis mice. Data are representative of two independent experiments.
